# Polish Expert Consensus on Metabolic and Bariatric Surgery: 2025 update

**DOI:** 10.20452/wiitm.2025.17950

**Published:** 2025-02-09

**Authors:** Piotr Major, Michał Orłowski, Piotr Małczak, Natalia Dowgiałło‑Gornowicz, Artur Binda, Paweł Bogdański, Andrzej Budzyński, Dorota Budzyńska, Michał Janik, Paweł Jaworski, Krzysztof Kaseja, Łukasz Kaska, Bartosz Katkowski, Tomasz Koszutski, Grzegorz Kowalski, Dominika Krakowczyk, Andrzej Kwiatkowski, Paweł Lech, Tomasz Lewandowski, Wojciech Lisik, Wojciech Makarewicz, Maciej Matyja, Wojciech Milanowski, Rafał Mulek, Piotr Myśliwiec, Lucyna Ostrowska, Maciej Pastuszka, Krzysztof Paśnik, Michał Pędziwiatr, Monika Proczko‑Stepaniak, Hady Razak Hady, Tomasz Rogula, Anna Różańska‑Walędziak, Jerzy Sieńko, Jacek Sobocki, Michał Spychalski, Jacek Szeliga, Tomasz Szewczyk, Michał Szymański, Paweł Szymański, Wiesław Tarnowski, Maciej Walędziak, Mateusz Wityk, Mariusz Wyleżoł, Michał Wysocki

**Affiliations:** 2nd Department of General Surgery, Jagiellonian University Medical College, Kraków, Poland; Department of General and Oncological Surgery, Ceynowa Hospital, Wejherowo, Poland; Department of General, Minimally Invasive and Elderly Surgery, Collegium Medicum, University of Warmia and Mazury, Olsztyn, Poland; Department of General, Oncological and Bariatric Surgery, Centre of Postgraduate Medical Education, Orłowski Hospital, Warszawa, Poland; Department of Treatment of Obesity, Metabolic Disorders and Clinical Dietetics, Poznan University of Medical Sciences, Poznań, Poland; Department of General Surgery and Surgical Oncology, Ludwik Rydygier Memorial Hospital in Krakow, Kraków, Poland; General Surgery Department, Military Institute of Aviation Medicine, Warszawa, Poland; Department of General Surgery and Transplantation, University Clinical Hospital no. 2 in Szczecin, Poland; Center for Surgical Treatment of Obesity, Independent Public Healthcare Center of the Ministry of Interior and Administration in Gdansk, Gdansk, Poland; Department of General and Vascular Surgery, St. John Paul II Specialist Medical Center, Polanica Zdrój, Poland; Department of Pediatric Surgery and Urology, School of Medicine in Katowice, Medical University of Silesia, Katowice, Poland; Department of General and Endocrine Surgery, Faculty of Medical Sciences in Zabrze, Medical University of Silesia, Katowice, Poland; Department of General, Oncological, Metabolic and Thoracic Surgery, Military Institute of Medicine – National Research Institute, Warszawa, Poland; Center of General, Bariatric and Oncological Surgery, Ełk, Poland; Department of General and Transplant Surgery, Medical Univeristy of Warsaw, Warszawa, Poland; Department of General Surgery with Division of Surgical Oncology, Specialist Hospital in Koscierzyna, Kościerzyna, Poland; Second Division of Radiology, Medical University of Gdansk, Gdańsk, Poland; Surgical Oncology Clinic, Maria Sklodowska‑Curie National Cancer Institute, Kraków, Poland; EuroMediCare Specialist Hospital and Clinic, Wrocław, Poland; First Department of General and Endocrine Surgery, Medical University of Bialystok, Białystok, Poland; Department of Dietetics and Clinical Nutrition, Medical University of Bialystok, Białystok, Poland; Department of General and Miniinvasive Surgery, Łęczna Hospital, Łęczna, Poland; Department of General, Gastroenterological and Oncological Surgery, Collegium Medicum of the Nicolaus Copernicus University, Poland; Department of General, Oncological and Transplant Surgery, University Medical Center, Medical University of Gdansk, Gdańsk, Poland; Second Clinical Department of General and Gastroenterological Surgery, Medical University of Bialystok, Białystok, Poland; DHR Health Bariatric & Metabolic Institute, Edinburg, Texas, United States; Institute of Medical Sciences of the Cardinal Stefan Wyszyński University, Warszawa, Poland; Institute of Physical Culture Sciences, University of Szczecin, Poland; Department of General Surgery and Clinical Nutrition, Centre for Postgraduate Medical Education, Warszawa, Poland; Department of General and Oncological Surgery, Medical University of Lodz, Łódź, Poland; Brzeziny Specialist Hospital, Brzeziny, Poland; Department of General Surgery, Specialist Hospital in Legnica, Legnica, Poland; Department of General and Oncological Surgery, Regional Health Centre, Lubin, Poland; Department of General, Vascular, and Oncological Surgery, Medical University of Warsaw, Warszawa, Poland

**Keywords:** bariatric surgery, metabolic surgery, pharmacological treatment of obesity, recommendations, surgical treatment of obesity

## Abstract

This document presents a comprehensive update to the Polish national recommendations on metabolic and bariatric surgery, developed by a panel of experts based on the latest clinical and scientific evidence. In light of a nearly 500% increase in the number of bariatric procedures in Poland since 2014 and significant technological advancements, it became necessary to revise national guidelines in line with international standards set by the International Federation for the Surgery of Obesity and Metabolic Disorders and the American Society for Metabolic and Bariatric Surgery. A pivotal development in Poland was the implementation of the Comprehensive Specialist Care in Bariatrics (KOS‑BAR) program in 2021, which ensured more accessible and safer care for patients with severe obesity through a structured and multidisciplinary approach.

The consensus covers the full scope of care: from the epidemiology of obesity, surgical eligibility criteria, and preoperative preparation, to the choice of surgical technique, postoperative care, management of complications, and revisional procedures. The document also includes guidance on pharmacological treatment as an addition or alternative to surgery, and addresses the needs of specific patient groups, such as pediatric patients, elderly individuals, women of reproductive age, and those with comorbid conditions.

The aim of this update is to provide Polish patients with access to safe, effective, and evidence‑based treatment for obesity, while also aligning the health care system with the growing public health challenge posed by the obesity epidemic.

## BACKGROUND

Metabolic and bariatric surgery (MBS) is one of the most rapidly developing sur‑ gical specialties in Poland. The latest data show an almost 500% increase in the number of bar‑ iatric procedures, as compared with 2014. This impressive progress reflects not only the epidemiological scale of obesity but also the dynamic development of this surgical field. Similar trends can be observed in reports from the International Federation for the Surgery of Obesity and Metabolic Disorders (IFSO) and the American Society for Metabolic and Bariatric Sur‑ gery (ASMBS), although the situation in Poland is particularly remarkable. Such growth would not have been possible without significant technological advancement in MBS over the past few decades. The complete dominance of minimally invasive techniques, introduction of surgeries with a favorable safety profile, and publication of numerous clinical studies con‑ firming undisputed postoperative benefits have led to the broad acceptance of MBS as a standard treatment for obesity. In Poland, a pivot‑ al step was the introduction of the Comprehensive Specialist Care in Bariatrics (KOS‑BAR) program in 2021, which ensured comprehensive, specialized care for patients with severe obesity. Thanks to the KOS‑BAR program, MBS has become more accessible and safer, significantly contributing to the increase in the number of performed procedures. This program has raised the standards of patient care and improved organization of the entire treatment process, from qualification for surgery to postoperative care.^1^ Since the publication of the MBS guidelines in 1991, bariatric surgery has undergone a complete transformation. As a consequence, it has become essential to review and update the original guidelines that for years served as a basis for patient qualification for weight loss surgery. Implementation of the new 2022 ASMBS and IFSO guidelines in Poland requires changes in national recommendations, which were last up‑ dated in 2017. Adapting these guidelines is crucial to ensure that Polish patients have access to the latest, proven, and safe methods for obesity treatment.^2^^,^^3^^,^^4^^,^^5^

## 1. INTRODUCTION

### 1.1 Outline of obesity epidemiology worldwide and in Poland

Obesity prevalence is rising both in developed and developing countries, reaching epidemic proportions. Over the past 30 years, the number of people with obesity has tripled worldwide. In the literature, the term globesity has emerged to vividly illustrate the scale of the problem. Obesity is no longer a condition solely associated with developed and wealthy countries. The in‑ crease has been observed in all countries glob‑ ally. Currently, the problem affects all socioeconomic groups. In 1998, the World Health Organization (WHO) recognized obesity as the most serious chronic health problem of the modern world. According to 2022 data, 1 in 8 people worldwide has obesity. Among adults, the problem of overweight affects 43% of the global population (up from 39% in 2014), while 16% suffer from obesity (up from 13% in 2014).

The highest percentage of individuals with obesity is found in the United States, where, according to the 2015 World Obesity Federation report, more than 60% of adults are over‑ weight, and more than half of those have obesity. Based on the “2015 Health at a Glance” re‑ port by the Organization for Economic Co‑operation and Development, the rate of obesity is growing fastest in populations with lower levels of education, particularly among women. According to Eurostat data from 2019, nearly 53% of the European population are overweight. The problem of overweight and obesity affects different regions of Europe to varying degrees, across various social groups, and among both men and women, yet the number of obese individuals is steadily increasing in all European countries.

In Poland, around 300 000 individuals have a body mass index (BMI) above 40 kg/m2, and in more than 1.5 million it exceeds 35 kg/m2. The obesity problem in Poland has been analyzed in projects such as the Pol‑MONICA, NATPOL PLUS, and Multicenter National Health Survey (WOBASZ). In the Polish population aged 15 and older, a BMI below 25 kg/m2 was found in only 43%, while 38% were overweight and 18.5% had obesity. According to the Central Statistical Office (GUS) data from 2019, this problem affected more than 65.1% of men (45.6% overweight, 19.5% obese) and almost 48.9% of women (31.3% overweight, 17.6% obese). It should be noted that, based on GUS data, there has been an increase in the prevalence of overweight and obesity in Poland: in 2009, excessive body weight affected 61% of men and 45% of women. Obesity is particularly hazardous to the youngest. Ex‑ cess weight in childhood and adolescence leads to obesity in adulthood. In Europe, overweight is the most common health issue in childhood. According to the latest WHO report from 2024, 37 million children under the age of 5 are overweight. Globally, over 390 million minors are overweight, with 160 million suffering from obesity; a sharp increase as compared with 2013, when the number of overweight children was estimated at around 42 million. Currently, about 20% of children in Europe are overweight, and nearly one‑third of them are obese. It is estimated that by 2030, 1 in 10 children will be obese. According to WHO data, the highest percent‑ age of overweight (36%) and obese (23%) children among 11‑year‑olds was found in Poland.^6^^,^^7^^,^^8^^,^^9^^,^^10^

### 1.2 Identifying the dangers associated with obesity: complications of obesity

Obesity leads to the development of complications stemming from:

- metabolic consequences;
- mechanical consequences;
- emotional consequences;
- social consequences.

Confirming the presence of obesity‑related complications in the preoperative period is crucial for patient qualification for MBS, selection of the surgical method, and assessment of the safety of the proposed surgical treatment.

The most common complications of obesity are:

- type 2 diabetes (T2D), including all stag‑ es preceding the full clinical manifestation of the disease, such as insulin resistance, abnormal fasting blood glucose, and impaired glucose tolerance;
- hypertension;
- lipid disorders, such as atherogenic dys‑ lipidemia, characterized by elevated levels of triglycerides, reduced levels of high‑density lipoprotein cholesterol, and the presence of abnormal small dense low‑density lipoprotein particles;
- fatty liver disease;
- gallstones;
- polycystic ovary syndrome;
- erectile dysfunction;
- gastroesophageal reflux disease (GERD);
- increased incidence of malignant tumors;
- degenerative changes in the musculoskele‑ tal system;
- sleep apnea syndrome;
- depression.

It is estimated that the overall number of obesity‑related complications may include around 200 different pathologies. The causes of the onset of most of these complications are complex, but obesity plays a crucial role in their development, as evidenced by their remission or an improvement in their course with weight reduction. Among patients qualifying for MBS, these complications are present in almost all individuals. The absence of such complications typically indicates an incomplete examination, rather than reflecting an actual health status of the patient or the extent of obesity. Most commonly, these complications manifest as multidisease syndromes, where obesity leads to the development of, for example, hypertension, T2D, and joint degeneration.

Obesity significantly shortens the life expectancy of patients. It has been shown that for individuals with a BMI ranging from 40 to 45 kg/m2, the expected lifespan is 8 to 10 years shorter than for those with a BMI between 22.5 and 25 kg/m2. This reduction in life expectancy is comparable to the effect of smoking.

The statistics related to causes of death associated with obesity may be distorted. In the case of most patients who die from obesity‑related complications (eg, T2D, hypertension, coronary artery disease, and many others), the underlying cause of these conditions—obesity—is of‑ ten not listed as the primary cause of death; instead, other diseases that are complications of obesity are typically considered.

When analyzing the impact of severe obesity on health and lifespan, it should not be forgotten that all the aforementioned complications significantly contribute to the reduced quality of life for individuals with obesity. Often, the disease is so debilitating that it renders patients completely disabled long before death. 

Currently, the treatment of obesity is primarily palliative, focusing on managing the com‑ plications of the disease rather than addressing its root causes. This approach to obesity and its consequences leads to ineffective management of financial resources allocated to health care. This issue becomes especially significant in MBS, given numerous scientific studies pointing to the high cost‑effectiveness of this treatment method for obese patients. In light of the rapid increase in the number of patients with obesity, it is crucial to effective‑ ly manage financial resources, accounting for obesity treatment as the basis for handling its complications.

### 1.3 Surgical treatment of obesity

Conservative treatment of obesity (diet, exercise, and other lifestyle interventions), particularly in the case of severe obesity, is characterized by low effectiveness and a lack of lasting results. In many cases, after an attempt at conservative treatment, obesity recurs, often exceeding the initial levels, leading to disease progression. Despite the introduction of more effective drugs in the pharmacotherapy of severe obesity, bar‑ iatric surgery remains the most effective meth‑ od of treatment. Surgery has ceased to be merely a treatment for excessive body weight. The more significant effects are the metabolic results of the procedure, which lead to the resolution of obesity‑related complications. This is confirmed by numerous studies documenting the superiority of surgical treatment of obesity over conservative methods, not only in terms of weight re‑ duction but also with respect to the resolution of diseases caused by obesity, such as diabetes, hypertension, lipid disorders, fatty liver disease, and the reduction of certain malignancy incidences. It has been proven that surgical treatment of patients with obesity extends their life and significantly improves its quality. Considering the benefits of surgical treatment for severe obesity and its complications, it should be regarded as the treatment of choice for the patients who meet the qualification criteria.^11^^,^^12^^,^^13^^,^^14^^,^^15^^,^^16^^,^^17^^,^^18^^,^^19^^,^^20^.

## 2. INDICATIONS FOR SURGICAL TREATMENT OF OBESITY

### 2.1 Body mass index

The qualification criteria for surgical treatment of severe obesity are based on the assessment of BMI, defined as a patient’s weight in kilograms divided by the square of their height in meters.

Indications for surgical treatment of severe obesity include:

- BMI of 35 kg/m2 or higher, regardless of the presence or absence of evident obesity‑related diseases;
- Surgical treatment of obesity may also be considered in patients with a BMI of 30–34.9 kg/m2 and T2D, if hyperglycemia persists despite the use of oral medications and insulin.
- Situations where obesity renders a patient in‑ eligible for an essential surgical procedure in an‑ other medical specialty (eg, orthopedic surgery, neurosurgery, hernia surgery, organ transplant). In such cases, MBS serves as a bridging therapy and should be considered to induce significant weight loss, thereby reducing the risk of the originally planned procedure.
- Patients who have previously undergone bariatric surgery but have not achieved the desired therapeutic effect (either in terms of weight reduction or resolution of obesity‑related diseases) should be offered revisional surgery.

The BMI criterion refers to the highest reported value in the past. Preoperative weight reduction that lowers BMI below the abovementioned thresholds is not a contraindication to surgical treatment.

A lack of confirmed weight loss attempts through conservative methods before an elective surgical treatment does not constitute a contraindication to surgery in adults.

Bariatric surgery as bridging therapy:

- Orthopedic procedures: patients with a BMI over 40 kg/m2 undergoing total hip or knee re‑ placement surgeries face an increased risk of com‑ plications, such as rehospitalization, surgical site infections, and deep vein thrombosis. MBS per‑ formed within 2 years before joint replacement significantly reduces the risk of these periopera‑ tive complications.
- Hernia surgery: obesity increases the risk of abdominal hernias and complications, such as infections, impaired wound healing, and recurrence after hernia repair. Patients with large abdominal wall hernias may benefit from a 2‑stage treatment: bariatric surgery as the first stage, followed by hernia repair, which can lower the risk of complications and recurrence.
- Transplantation: obesity may limit patient eligibility for organ transplant, serve as a relative contraindication, and pose significant intraoperative technical challenges. MBS as a bridging therapy can significantly improve transplant eligibility for patients with kidney or liver failure. Additionally, weight loss following MBS can increase eligibility for heart transplant. In some cases, bar‑ iatric surgery–induced weight loss can improve left ventricular ejection fraction, potentially re‑ ducing the need for transplant.^21^^,^^22^^,^^23^^,^^24^^,^^25^^,^^26^^,^^27^^,^^28^^,^^29^^,^^30^^,^^31^^,^^32^^,^^33^^,^^34^^,^^35^^,^^36^.

### 2.2. Age criteria

Bariatric surgery is recommended for patients aged 18 to 65 years.

#### 2.2.1. Age over 65 years

MBS is not contraindicated in individuals over 65 years of age, and there is currently no upper age limit for obesity surgery. Recent studies indicate that these procedures may be associated with a higher incidence of postoperative complications, especially early complications, as compared with procedures performed in younger populations— this is due to multimorbidity rather than age itself. However, MBS still provides significant benefits, including excess weight loss and remission or alleviation of obesity‑related diseases.^37^ MBS in patients over 65 years of age may not necessarily extend life expectancy, but it significantly improves quality of life.

When qualifying elderly patients for bariatric surgery, chronological age should not be the primary factor. Instead, physiological changes associated with aging, such as frailty, cognitive abilities, obesity‑related diseases, and cardiopulmonary fitness should be considered, as they influence postoperative outcomes and complication risks.

Additionally, it is essential to recognize that the likelihood of remission or improvement in obesity‑related diseases decreases with time. There‑ fore, especially in this age group, patients should be qualified for surgery as soon as possible after necessary dietary and psychological preparation and optimization of chronic disease management.^38^^,^^39^^,^^40^^,^^41^^,^^42^^,^^43^^,^^44^^,^^45^^,^^46^^,^^47^.

#### 2.2.2. Age under 18 years

MBS is a safe and effective treatment for obesity in children and adolescents. Furthermore, the remission of obesity‑related diseases in this age group is effective and long‑lasting. MBS in individuals under 18 years of age is more effective than family‑based therapy or behavioral interventions alone, and the decision for surgery should not be postponed until the patient reaches adulthood. Surgical qualification of patients aged under 18 years of age should be performed in multidisciplinary centers following strict criteria:

- BMI greater than 40 kg/m2 (or 140% of the 95th percentile for age and sex);
- BMI greater than 35 kg/m2 (or 120% of the 95th percentile) with at least 1 obesity‑related comorbidity (eg, hypertension, T2D, glucose intolerance or insulin resistance, dyslipidemia, obstructive sleep apnea, left ventricular hypertrophy, GERD, orthopedic conditions related to obesity, idiopathic intracranial hypertension, metabolic dysfunction–associated steatotic liver disease (MASLD), significant psychological disorders);
- No requirement for skeletal maturity (MBS in younger patients does not impair growth; in fact, weight loss after surgery may improve growth) or sexual maturity;
- Informed consent from both the patient and their guardian, ensuring an understanding of the procedure and the lifelong commitment to compliance, including vitamin supplementation, a high‑protein diet, and regular follow‑up care;
- The child’s willingness to undergo bariatric surgery;
- A preoperative preparation period of approximately 6 months (although part of Polish pediatric obesity societies recommend a 12‑month preparation period);
- Weight loss before surgery, or at least no weight gain during the preparation period, is recommend‑ ed. Maintaining or reducing body weight confirms the patient’s motivation for treatment and willingness to change previous habits.
- Intellectual disability is not an absolute contra‑ indication to bariatric surgery.^48^^,^^49^^,^^50^^,^^51^^,^^52^^,^^53^

### 2.3. Contraindications to metabolic and bariatric surgery

#### Absolute contraindications

- Incurable diseases leading to cachexia (eg, active cancer, AIDS);
- Conditions that are life-threatening in the short term (eg, recent myocardial infarction, severe chronic obstructive pulmonary disease);
- Endocrine disorders causing obesity (eg, Cushing syndrome);
- Severe coagulation disorders;
- Lack of patient cooperation or refusal to accept surgical outcomes due to:
  - active alcohol or drug addiction; qualification for MBS may be considered after at least 1 year of documented abstinence;
  - uncontrolled mental illness despite treatment and pharmacotherapy;
  - severe intellectual disability;
- Inability to participate in continuous long-term follow-up after surgery;
- The 12-month period preceding a planned pregnancy, current pregnancy, and breastfeeding (until delivery and the end of lactation); pregnancy is no longer contraindicated after a period 24 months since the date of MBS;
- Lack of patient consent or uncertainty about the appropriateness of surgical treatment.^23^^,^^54^^,^^55^

### 2.3.2 Relative contraindications

- Weight gain directly before surgery, indicating a lack of patient compliance;
- Active peptic ulcer disease requiring treatment before surgery; in the case of asymptomatic Helicobacter pylori infection, eradication be‑ fore surgery is recommended but not absolutely necessary;
- Undiagnosed and untreated portal hypertension due to liver cirrhosis.^23^^,^^54^^,^^55^

## 3. PREOPERATIVE ASSESSMENT AND PREPARATION FOR BARIATRIC SURGERY

### 3.1. Main objectives of preoperative assessment and preparation for bariatric surgery

Overview of surgical treatment for obesity:

- Informing the patient about the benefits of surgical treatment;
- Providing detailed information on perioperative risks, complications, and alternative treatment methods, both verbally and in a written form through an informed consent document;
- Presenting the limitations associated with surgical treatment and its effects, particularly the necessity of adhering to dietary recommendations;
- Securing patient acceptance of lifestyle changes resulting from surgical treatment and the necessity of continuous cooperation with the medical team (including dietary habit modifications, smoking cessation, and physical activity);
- Optimizing the treatment of obesity‑related diseases and other comorbid conditions to reduce perioperative risk;
- Assessing the patient’s motivation level and ability to cooperate during the perioperative period, as well as in long‑term follow‑up.^56^^,^^57^^,^^58^.

### 3.2. Multidisciplinary team involved in bariatric patient care

Patient qualification and preparation for bariatric surgery should be conducted by a multidisciplinary team consisting of specialists from various fields. The objective of the team is to prepare a patient for surgery and oversee appropriate and complication‑free weight loss both in the early and long‑term postoperative periods.

A key role in the multidisciplinary team is played by the coordinator, who supervises the preparation process and the entire treatment course. This should be a physician experienced in treating and caring for patients with severe obesity, preferably a bariatric surgeon.

The best bariatric treatment outcomes are achieved when the multidisciplinary team includes the following specialists:

- a bariatric surgeon;
- an internal medicine specialist;
- an anesthesiologist;
- a nutritionist;
- a psychologist / psychiatrist;
- a care coordinator.

If needed, specialists from other medical disciplines experienced in bariatric patient care can be consulted, such as a cardiologist, pulmonologist, diabetologist, endocrinologist, physiotherapist, and others, depending on patient needs.^56^

### 3.3. Scope of preoperative assessment

All patients qualifying for surgical obesity treatment should be assessed for the severity of obesity‑related diseases and other comorbidities, particularly those affecting surgical eligibility. A proper evaluation should include medical history taking, physical examination, psycho‑ social assessment, laboratory and imaging tests, and perioperative risk assessment. The results should be recorded in medical documentation.

The optimal scope of preoperative assessment should include:

- Health and nutritional status, obesity‑relat ed diseases;
- Body weight, BMI;
- Laboratory tests, such as:
  - complete blood count;
  - nutritional status parameters (lipid pro‑ file, total protein, albumin, iron, ferritin, vita‑ min B_12_, vitamin D_3_);
  - kidney function assessment (creatinine, urea);
  - liver function assessment (alanine aminotransferase, aspartate aminotransferase, γ‑glutamyl transferase);
  - carbohydrate metabolism assessment (fasting glucose, glycated hemoglobin [HbA_1c_]);
  - coagulation parameters;
  - endocrine function (thyroid‑stimulating hormone; expanded testing if an endocrine cause of obesity is suspected);
- Upper gastrointestinal endoscopy (evaluation of esophageal, gastric, and duodenal mucosal pathology, hiatal hernia, and H. pylori infection) for all patients regardless of symptoms;
- Abdominal ultrasound or, in selected cases, computed tomography scan (evaluation of abdominal abnormalities, particularly gallstones and MASLD);

- Electrocardiogram and, in selected cases, echocardiography (for preoperative cardiovascular evaluation and perioperative risk assessment in cardiac disease patients);
- Chest X‑ray (for general anesthesia suitability evaluation);
- Spirometry in selected cases (evaluation of respiratory reserve and potential obstruction);
- Psychological and dietary assessment of eating disorders (eg, compulsive eating), understanding of postoperative life changes, informed consent, and perioperative cooperation;
- Anesthesiology consultation (preoperative assessment of potential intubation difficulties);
- If needed, a dental consultation (oral health assessment);
- Polysomnography (screening for obstructive sleep apnea in the case of a positive STOP‑BANG questionnaire result).

Depending on the spectrum of comorbid conditions and results of the preoperative assessment, the extent of diagnostic tests may need to be expanded.^59^^,^^60^^,^^61^^,^^62^^,^^63^.

### 3.4. Duration and number of consultations in the pre‑ operative period

The best treatment outcomes are achieved when the preoperative preparation period lasts at least 3 months. The optimal period is consid‑ ered to be 6–12 months. During this time, at least 3 appointments with the bariatric care coordinator or other multidisciplinary team members are recommended.

### 3.5. Additional recommendations

During preoperative preparation, addition‑ al requirements should be presented to the patient to optimize outcomes:

- Significant weight reduction before surgery;
- If the left lobe of the liver is significantly en‑ larged due to metabolic dysfunction–associated steatohepatitis, a diet aimed at reducing liver volume should be introduced preoperatively.
- Due to an increased risk of peri‑ and postoperative complications, smoking cessation at least 6 weeks before surgery is recommended.
- In patients with a history of alcohol dependence, a documented abstinence period of at least 12 months is required. The risk of relapse post–bariatric surgery should be considered.
- Physical activity and exercise may improve fit‑ ness and respiratory efficiency; however, there is currently insufficient evidence supporting its routine recommendation before MBS.^64^^,^^65^

### 3.6. Preparation of patients under 18 years of age

The qualification and preparation of patients under 18 years of age should be conducted exclusively in collaboration with a multidisciplinary team experienced in pediatric bariatric surgery. The team should include:

- a pediatrician;
- a pediatric surgeon;
- a nutritionist;
- a psychologist or mental health specialist.

In this age group, in addition to the previously mentioned recommendations, preoperative assessment should also include an evaluation of the patient’s psychological maturity in terms of their ability to provide informed consent, willingness to undergo treatment, adherence to postoperative recommendations, and awareness of the limitations and consequences of surgery.

At every stage of surgical preparation of patients under 18 years of age, family involvement is obligatory. It is crucial to obtain family members’ acceptance and confirm their willingness to support surgical treatment, ensuring they understand the consequences of surgery and provide postoperative support. Informed consent for surgery must be signed by both the child and their legal guardian. Patient and family education should cover the types of MBS, as well as potential short‑ and long‑term complications.

The average preparation period should be approximately 6 months, during which, in addition to diagnostic procedures, education on healthy eating and lifestyle habits should be provided. Gradual dietary habit changes not only facilitate weight reduction but also contribute to faster acceptance and implementation of postoperative recommendations.^48^^,^^49^

## 4. SURGICAL TREATMENT

Systematic scientific research has led to a better understanding of the mechanisms of weight reduction and improved control of obesity‑related diseases in patients undergoing surgical treatment for severe obesity. Based on current knowledge, the previous classification of procedures into restrictive and malabsorptive surgeries appears to be outdated. Today, MBS is recognized not only for its weight loss effects (bariatric effect) but also for its capacity for improving obesity‑related conditions, particularly T2D (metabolic effect). Modern research demonstrates that these effects are inter‑ woven, and the impact of different surgical procedures on the body is far more complex than initially assumed. Therefore, classifying procedures solely based on these 2 categories does not fully capture the complexity of their mechanisms.

### 4.1. Types of metabolic and bariatric surgery

A variety of bariatric procedures are currently applied, each associated with different levels of difficulty, expected outcomes, and potential com‑ plications. New methods and modifications of existing procedures continue to emerge, requiring comparative studies and medium‑ to long‑term outcome evaluations before being recognized as safe, effective, and recommended by scientific and ethical committees.

According to the latest recommendations of leading bariatric surgery societies, including IFSO and ASMBS, the recognized metabolic and bariatric procedures include:

- sleeve gastrectomy (SG);
- Roux‑en‑Y gastric bypass (RYGB);
- one anastomosis gastric bypass (OAGB);
- single anastomosis duodeno‑ileal bypass with sleeve gastrectomy (SADI‑S);
- biliopancreatic diversion with duodenal switch (BPD‑DS).

According to a 2024 report on global trends in MBS for the years 2020–2021, SG and RYGB remain the most commonly performed bariatric and metabolic procedures worldwide. These surgeries offer the most favorable risk‑benefit ratio, as demonstrated by numerous long‑term follow‑up studies.

In recent years, OAGB has also gained recognition as a standard metabolic procedure and is now the third most commonly performed bariatric surgery globally. Another increasingly popular procedure is SADI‑S, which, according to IFSO and ASMBS, is a fully recognized MBS. SADI‑S is a modified version of BPD‑DS.

Recently, innovative surgical procedures have been gaining attention for their potential to facilitate weight loss and alleviate obesity‑related conditions. These procedures are currently under evaluation by scientific societies and await official recognition as standard MBSs. To receive broad recommendations, they require reliable medium‑ and long‑term studies to assess their safety, effective‑ ness, and associated risks. These include:

- SG with simultaneous fundoplication (sleeve fundoplication procedure);
- surgeries involving transit bipartition:
  - single‑anastomosis sleeve‑ileal bypass (SASI bypass);
  - single‑anastomosis sleeve‑jejunal bypass (SASJ bypass);
  - sleeve gastrectomy with transit bipartition (gastric bipartition; Santoro procedure);
- ileal transposition;
- gastric plication.

Since the Chapter for Metabolic and Bariatric Surgery of the Association of Polish Surgeons is a part of IFSO, Poland follows the IFSO guide‑ lines outlined in the document “Innovative Bar‑ iatric Procedures and Ethics in Bariatric Surgery: the IFSO Position Statement.” When proposing these procedures to patients, the surgeon must:

- Inform the patient that the procedure is not yet widely recognized as a standard method;
- Present the expected benefits and risks, empha‑ sizing the lack of long‑term comparative studies with established procedures;
- Disclose the surgeon’s experience with the pro‑ posed method;
- Offer alternative, recognized treatment options, allowing the patient to make an informed decision;
- Ensure long‑term postoperative care.

It is recommended that these procedures be performed within the framework of clinical trials, with prior approval from an ethics committee. This requirement does not apply to revision or emergency surgeries, where intraoperative decisions are dictated by the patient’s specific anatomical conditions. In such cases, the procedure

is not classified as experimental or innovative surgery.

Adjustable gastric banding (AGB), once a widely used method, is now considered historical and is no longer recommended. Approximately 20% of patients require band removal due to complications or insufficient bariatric effects.

Surgical procedures that are no longer per‑ formed include:

- biliopancreatic diversion (BPD) by Scopinaro;
- vertical banded gastroplasty (VBG);
- jejuno‑ileal bypass;
- vagal nerve blockade.

These procedures have been discontinued in favor of safer and more effective surgical options. However, patients who previously under‑ went these operations may require further treat‑ ment for obesity and metabolic disorders.2,66 ‑70

### 4.2. Patient qualification for specific bariatric procedures

There are currently no uniform guidelines for selecting a specific bariatric procedure for an individual patient. Different bariatric procedures vary in their mechanisms, expected bariatric and metabolic effects, potential complications, and long‑term care requirements. The choice of the optimal surgical method for each patient should be individualized and consider the primary treatment goal (bariatric vs metabolic effect).

When selecting a treatment method, the following factors should be considered:

- Experience of the surgeon and bariatric center;
- Patient’s BMI;
- Age and overall health status;
- Obesity‑related diseases, particularly T2D;
- Anatomical changes in the gastrointestinal tract due to previous surgery or medical conditions (eg, hiatal hernia, prior gastric or intestinal resections);
- Eating habits;
- Patient’s expectations and preferences;
- Patient’s ability to adhere to perioperative and long‑term follow‑up care.

Although there is no definitive scientific consensus, most experts recommend:

- SG for patients at a high surgical risk or older adults (>65 years old);
- RYGB rather than SG in the cases of large hiatal hernia and / or GERD or Barrett esophagus;
- RYGB, OAGB, or SADI‑S rather than SG for patients with severe, insulin‑dependent T2D;
- SADI‑S as a second‑stage procedure for patients with BMI >50 kg/m2 who did not achieve sufficient weight loss after previous SG.

A multidisciplinary team plays a crucial role in preparing patients for surgery by reducing modifiable perioperative risk factors. The final decision regarding surgical qualification, procedure selection, and patient readiness rests with the surgeon.

Due to the chronic nature of obesity and its multifactorial pathogenesis, MBS alone may be insufficient to achieve the desired outcomes in terms of weight loss and resolution of obesity‑related diseases. Patients with obesity require long‑term care in the postoperative period, during which additional surgeries or implementation of pharmacological treatment (adjuvant therapy) may be necessary to achieve the expected results.^3^^,^ ^23^^,^^71^

### 4.3. Recommendations regarding surgical technique

It is recommended that bariatric procedures be performed in centers with experience in MBS, by skilled staff, and with necessary equipment. For all types of surgical procedures, the surgeon’s and the team’s the level of experience appears to be crucial. The bariatric center should have the capacity to manage complications of bariatric surgeries, both in terms of the team’s skills and the availability of appropriate equipment.

The preferred approach for bariatric procedures is laparoscopy, which is associated with a lower complication rate and reduced perioperative mortality. If technical difficulties preclude performing the procedure laparoscopically or if it cannot be safely continued, it is advised to refrain from performing the surgery. Conversion to open surgery is not recommended. Addition‑ ally, in the treatment of complications, the preferred method should be minimally invasive, with conversion reserved for exceptional situations. 

If a patient would benefit most from a procedure not available at the current center, they should be referred to a referral center that can offer the optimal form of treatment.

It is essential to precisely document the de‑ tails of the surgical procedure based on a standardized protocol, including elements such as:

- Diameter of the nasogastric tube used during the procedure;
- Type of anastomosis;
- Materials used (number and type of staplers, sutures, suturing of anastomosis lines);
- Length of individual intestinal loops;
- Information about the closure of mesenteric defects;
- Documentation of any histopathologic examination of a surgical specimen.^23^^,^^72^

### 4.4. Recommendations regarding perioperative management

According to available scientific data, the use of a structured perioperative management proto‑ col contributes to improved short‑ and long‑term treatment outcomes.

According to the Enhanced Recovery After Surgery (ERAS) Society guidelines, the most important elements in perioperative management of patients undergoing bariatric surgery are:

- Preparing the patient before surgery (cessation of smoking and alcohol consumption, increasing physical activity, modifying dietary habits, pre‑ operative weight loss);
- Reducing fasting time (administering a carbohydrate‑rich drink 2–3 hours before surgery);
- Balanced fluid therapy (limiting intravenous fluid administration, using balanced crystalloids, early initiation of oral fluid intake);
- Perioperative antibiotic prophylaxis;
- Thromboprophylaxis (both mechanical meth‑ ods and low‑molecular‑weight heparin, with dos‑ age tailored individually);
- Routine abdominal drainage appears to be sci‑ entifically unfounded;
- Nasogastric tube should not be left in place;
- Postoperative passive oxygen therapy (using appropriate equipment / continuous positive air‑ way pressure in patients with diagnosed obstruc‑ tive sleep apnea);
- Multimodal analgesia with limited opioid use, recommended use of local anesthesia at the surgical site and regional blocks;
- Multimodal antiemetic therapy for postoperative nausea and vomiting;
- Early mobilization of the patient after surgery;
- Early introduction of oral nutrition, oral fluid intake as early as on the day of surgery.^56^^,^^73^

## 5. ENDOSCOPY IN BARIATRIC SURGERY

### 5.1 Gastroscopy in qualification for surgical treatment

Gastroscopy (GFS) is an essential element in the qualification process for bariatric treatment, as it allows direct assessment of the mucosa of the esophagus, stomach, and duodenum. Patients should be referred for this examination regardless of the presence of symptoms. GFS enables the diagnosis (according to the literature, in 55% of cases) of conditions such as hiatal hernia, changes in the esophageal mucosa resulting from GERD, Barrett esophagus, peptic ulcers of the stomach and duodenum, gastric and duodenal mucosal inflammation (H. pylori infection), portal hypertension effects (portal gastropathy, esophageal / gastric varices), gastrointestinal stromal tumors, precancerous lesions and malignant tumors of the esophagus / stomach. According to the available literature, this examination should be performed in every patient qualified for MBS. It allows for diagnosis and enables biopsy‑based verification of lesions and the initiation of appropriate treatment be‑ fore planned surgery. The endoscopic image can facilitate the choice of the proper obesity treat‑ ment method; up to 25% of GFS results lead to delayed surgery or a change in the proposed pro‑ cedure type. The primary factors influencing a change in surgical strategy after preoperative GFS are the presence of Barrett esophagus, in‑ flammatory changes typical of GERD, large hia‑ tal hernia, and mucosal atrophy with intestinal

metaplasia in the antrum.

Recommendations:

- Every patient qualified for bariatric surgery should undergo GFS.
- The result of preoperative GFS examination should be one of the factors determining the type of bariatric surgery.^74^^,^^75^

### 5.2 Gastroscopy in postoperative endoscopic surveil‑ lance after bariatric procedures

Due to an increased risk of developing pathologies at the esophagogastric junction during the postoperative period, we recommend the following approach, based on the IFSO and ASMBS guidelines:

- For patients undergoing SG and OAGB, GFS should be performed 12 months after surgery, and every 3–5 years thereafter.
- For other cases, GFS should be performed in the presence of clinical symptoms.

### 5.3 Endoscopic treatment of obesity

In recent years, several methods of endoscopic treatment for obesity have been developed. Many of these did not go beyond clinical trials with small patient groups, and some methods have been withdrawn due to a risk of complications or insufficient procedural outcomes. Currently, 2 types of endoscopic procedures are routinely performed in obesity treatment: gastric balloon implantation and endoscopic gastroplasty. These methods have proven efficacy, a low com‑ plication risk, and provide a valuable alternative or complement to classic surgical obesity treatments. It should be emphasized that performing bariatric endoscopic procedures should be pre‑ ceded by proper training that covers the practical aspects of conducting such surgeries, as well as the pathophysiology of obesity, modern pharmacotherapy, and surgical treatment. First bar‑ iatric endoscopic procedures must be performed under the supervision of an expert. To ensure optimal therapeutic results, individuals treated with endoscopic obesity management methods should be supported by a multidisciplinary team, similarly to the surgically operated patients.^75^

#### 5.3.1. Gastric balloons: targeted and bridge therapy

Gastric balloons have been used for obesity treatment for over 30 years. Currently, the European market offers implants registered for 6 and 12 months of therapy. Depending on the type of balloon and its inflation level, the expected therapeutic effect at the end of the treatment is 10% to 15% of total body weight loss (TWL). The com plication risk reported in most meta‑analyses does not exceed 2%. However, it should be noted that in the event of gastrointestinal obstruction or gastric perforation, these complications can be severe and lead to patient death. A rare complication associated with gastric balloon therapy is acute pancreatitis. In the case of confirmed acute pancreatitis, urgent balloon removal is advised. Most patients report nausea / vomiting and other dyspeptic symptoms during the early stages of therapy, which, in extreme cases, may require early removal of the implant. The balloon removal procedure should be performed under general anesthesia with endotracheal intubation due to a risk of aspiration of food contents into the airways. A major issue in treating obesity with a gastric balloon is the recurrence of the condition after balloon removal. It should be emphasized that the support of a multidisciplinary team is necessary for achieving satisfactory treatment results. According to the European Society of Gastrointestinal Endoscopy (ESGE) guidelines, performing gastric balloon implantation procedures independently should be preceded by 10 procedures conducted under the super‑ vision of an expert.

Indications for gastric balloon treatment include:

- Patients with overweight and obesity‑related comorbidities, in whom pharmacological treatment and lifestyle modifications have not yielded satisfactory results;
- Patients with grade I obesity, in whom pharmacological treatment and lifestyle modifications have not yielded satisfactory results;
- Patients with grade III obesity, as a bridge to definitive surgical treatment;
- In the cases of patients with grade II obesity who refuse surgical treatment and prefer endoscopic treatment, endoscopic gastroplasty should be considered due to its greater efficacy and long‑term outcomes.^76^^,^^77^^,^^78^

#### 5.3.2 Endoscopic gastroplasty

This endoscopic procedure involves reducing the stomach volume by performing plication of its wall with sutures encompassing the full thick‑ ness of the wall in the stomach body region, from the angle to the upper part of the body, without performing fundoplasty. This reduces the organ’s volume by about 70%. In addition to the restrictive effect, another benefit of endoscopic gastroplasty (ESG) is the 2–3 times slower passage through the stomach, which results in prolonged satiety. Results from numerous studies indicate a high efficacy of this method in treating obesity and its complications. Reported efficacy in terms of reducing TWL is around 16%–18%. In the multicenter, prospective MERIT study, the beneficial effect of ESG on the treatment of hypertension and T2D was demonstrated. Unlike the gastric balloon, gastric plication is designed to provide permanent volume reduction. Avail‑ able data indicate stable weight loss in 5‑year follow‑up. The risk of complications after ESG is low, around 1%–2%. The most common com‑ plication is gastrointestinal bleeding. Less frequent complications include gastric perforation, inflammatory infiltration / perigastric fluid collection, and pneumothorax.

In the case of an ESG failure, MBS may be per‑ formed. There is no increased complication rate in this patient group. Due to a potential risk of interaction between the stapler and sutures used during ESG, it is recommended to remove the sutures before performing the surgical procedure. 

According to the ESGE guidelines, performing ESG independently should be preceded by 20 procedures under expert supervision. Con‑ firmed efficacy and a favorable safety profile were the basis for including ESG in the 2022

IFSO and 2024 National Institute for Health and Care Excellence (NICE) guidelines. Indications for ESG include:

- Patients with overweight and obesity‑related diseases, in whom pharmacological treatment and lifestyle modifications have not yielded satisfactory results;
- Patients with grade I and II obesity, including those with T2D, in whom pharmacological treatment and lifestyle modifications have not yielded satisfactory results;
- Patients with grade III obesity who are not suitable candidates for surgical treatment or re‑ fuse such treatment.

Current studies are focused on combined methods for treating obesity, including induction pharmacological therapy with glucagon‑like peptide 1 (GLP‑1) analogs and the ESG procedure. While there is no standardization of this treatment approach, preliminary results are encouraging.

Endoscopic gastric suturing procedures are also possible as a revision procedure after laparoscopic SG and RYGB. Five‑year follow‑up results are promising, particularly in the RYGB patient group, where surgical re‑intervention is especially challenging. TWL in this group was 8.8%.^79^^,^^80^^,^^81^^,^^82^^,^^83^^,^^84^^,^^85^

### 5.4 Application of endoscopy in the treatment of surgical complications

Endoscopic procedures are the standard in the diagnosis and treatment of MBS complications. Effective endoscopic treatment is possible with a significant experience in postoperative upper gastrointestinal tract anatomy, fluorosco‑ py‑guided procedures, and proficiency in a wide range of endoscopic techniques. Treatment should be performed in close cooperation with the surgical team, with the option to combine classic surgical methods (re‑laparoscopy / drain‑ age). Recommendations for endoscopic treatment of the most common MBS complications are discussed below.

### 5.4.1 Treatment of anastomotic stricture after Roux‑en‑Y gastric bypass

Diagnosis is based on dysphagia symptoms reported by the patient, in combination with endoscopic imaging (inability to pass a standard gastroscope through the gastrojejunostomy). In some cases, radiological imaging involving oral administration of a contrast agent may also be helpful. Treatment of strictures mainly involves balloon dilation to a diameter of 10–15 mm. Achieving a durable effect often re‑ quires more than 1 dilation session. In recurrent stricture cases, steroid injection after balloon dilation may be considered. Attention should be paid to the diameter of the balloon used. Excessive dilation is associated with a risk of perforation, and may decrease the bariatric effect of RYGB.^86^^,^^87^

### 5.4.2. Endoscopic treatment of gastro‑gastric fistulas after Roux‑en‑Y gastric bypass

In the case of small fistulas, with a diameter up to 1 cm, endoscopic methods may be effective. The most common methods are over‑the‑scope (OTS) clips and endoscopic suturing. However, their effectiveness in chronic fistulas with a wider tract is limited. In the cases of failure, the treatment of choice is surgical revision.

Endoscopic treatment is effective and, in most cases, sufficient to close a gastro‑gastric fistula after endoscopic treatment of choledocholithiasis using the endoscopic ultrasound (EUS)‑directed transgastric endoscopic retrograde cholangiopancreatography (EDGE) method. This method allows access to the Vater papilla with a standard duodenoscope after performing an endoscopic gastro‑gastric anastomosis with a lumen‑appos‑ ing metal stent under EUS guidance.^88^^,^^89^

### 5.4.3. Treatment of leaks after sleeve gastrectomy

A leak in the staple line after SG is a serious complication, occurring in up to 5% of all SG procedures. Leaks are classified as early, within 7 days of surgery, and late, after 7 days. The most frequent location of a leak is the upper part of the staple line, near the gastroesophageal junction. Endoscopic treatment of leaks involves the use of self‑expanding stents, internal endoscopic drainage, and vacuum therapy. For late leaks, techniques that involve closing fistulas using OTS clips and endoscopic suturing kits may also be applicable. Self‑expanding stents are widely used in the treatment of leaks, including fully covered stents designed for bar‑ iatric patients. The average duration of therapy is 6–8 weeks. The treatment success rate is estimated at around 75%. Stent placement is often preceded by re‑laparoscopy and abscess drainage near the leak site. The most common complication after stent placement is migration, occur‑ ring in approximately 15% of cases. In the case of stent migration, repositioning under endoscopic control is necessary, and if stent migrates into the jejunum, surgical treatment is required. To prevent migration, attempts are made to fix the stent using clips and sutures placed endoscopically, but there is no evidence supporting their routine use. In the case of concomitant stenosis at the gastric angle, appropriate treatment is necessary (most often balloon dilatation to 30 mm).

A second effective method for treating leaks after SG is endoscopic vacuum therapy using exchangeable sponges. The vacuum therapy set is applied to the leak site and connected to a continuous suction system with a pressure of 75–120 mm Hg. The sponge needs to be replaced every 2–3 days. The treatment duration is generally shorter than with self‑expanding stents (approximately 3 weeks), with a success rate of around 90%.^90^^,^^91^^,^^92^^,^^93^.

#### 5.4.4 Treatment of gastrointestinal bleeding after metabolic and bariatric surgery

Gastrointestinal bleeding after MBS is relatively rare, with an estimated risk of around 1%. It occurs more frequently after RYGB and OAGB. Bleeding from the staple line after SG typically occurs into the peritoneal cavity. The most common time of bleeding occurrence is with‑ in 1–2 days of surgery. Regardless of the type of bariatric surgery, if bleeding symptoms occur, the treatment of choice is endoscopic intervention. The typical bleeding sites after RYGB and SG are the gastrojejunal anastomosis and the staple line, respectively. Endoscopic techniques of choice for bleeding treatment include mechanical methods (through‑the‑scope endoscopic clips), often combined with adrenaline injections. In the case of active bleeding, the endoscopic procedure should be performed under general endotracheal anesthesia. The endoscopic team performing the procedure should be capable of using at least 2 methods of hemostasis. Performing the endoscopic procedure early in the postoperative period does not increase the risk of anastomotic leaks and does not affect long‑term treatment outcomes.^94^^,^^95^

## 6. POSTDISCHARGE RECOMMENDATIONS AND LONG‑TERM FOLLOW‑UP

Metabolic and bariatric treatment does not end when the patient is discharged from the hospital. Patients require regular follow‑up as part of ongoing care provided by specialists familiar with the issues faced by individuals after MBS. 

The goal of continuous monitoring and long‑term care for patients after bariatric surgery is not only to improve outcomes in terms of weight loss but also to prevent long‑term com‑ plications and deficiencies. During subsequent stages of follow‑up, continuous patient education regarding proper nutrition is necessary.

To effectively monitor both short‑ and long‑term outcomes of bariatric treatment, it is essential to implement a national registry for bariatric surgeries, similar to the Metabolic and Bariatric Surgery Quality Improvement Program (MB‑SQIP) operating in the United States.^23^

### 6.1 Hospital discharge

According to the ERAS guidelines, a patient may be discharged home after bariatric surgery when the following criteria are met:

- Patient tolerates oral diet and consumes at least 1000 ml of fluids per day;
- Patient does not require intravenous fluid administration;
- Postoperative pain is manageable with oral medications;
- Patient’s physical activity level is similar to the presurgery level;
- After discharge, patient will remain under the care of a third party and will have access to the treating center, if necessary;
- There are no complications that would require rehospitalization.

Recommendations at discharge:

- Thromboprophylaxis with low‑molecular‑weight heparin for 14 days postsurgery;
- Proton pump inhibitors for at least 1 month postsurgery; some patients may require extend‑ ed use for up to 6 months or longer;
- Supplementation with vitamins and micro‑ and macronutrients is necessary in all patients, and it should be maintained in the long term in those who have undergone malabsorptive surgeries (eg, RYGB, OAGB, BPD, SASI, SADI‑S);
- Ursodeoxycholic acid 50–600 mg daily for 6 months.

Before discharge, the patient should be in‑ formed that:

- Excessive meal volume may lead to postoper‑ ative complications and unsatisfactory weight loss results;
- Adequate daily protein intake is essential to prevent malnutrition; 35% of dietary energy should come from 60–80 g of protein per day or –1.5 g/kg of ideal body weight;
- Diet rich in simple sugars and fats (eg, sweets, sugary sodas) should be avoided; this also helps prevent dumping syndrome;In the first 6–8 weeks, oral medications should, if possible, be taken in crushed or liquid form; the pharmacokinetics of medications in bariat‑ ric patients may change, affecting absorption, distribution, metabolism, and / or elimination. There is insufficient data to make general dos‑ ing recommendations, so individual risk assess‑ ment is necessary.
- In the first 6–8 weeks, oral medications should, if possible, be taken in crushed or liquid form; the pharmacokinetics of medications in bariatric patients may change, affecting absorption, distribution, metabolism, and / or elimination. There is insufficient data to make general dosing recommendations, so individual risk assessment is necessary.

Regular physical activity has proven benefits after MBS.

All patients should receive instructions on how to manage complications. The center performing MBS should provide 24‑hour emergency access for patients who develop sudden health issues related to the surgery. If complications arise, treatment should ideally be carried out in centers with experience in MBSs.^54^,^56^,^96^,^97^,^98^,^99^,^100^,^101^,^102^

### 6.2 Specialists involved in post–metabolic and bar‑ iatric surgery care

Long‑term follow‑up for bariatric surgery patients should be managed by a specialist multidisciplinary team. The follow‑up program is coordinated by a program coordinator, such as a specialist nurse or, in certain cases, a surgeon or internal medicine specialist (eg, endocrinologist). Depending on patient needs, access to specialists involved in the preoperative qualification process for surgeries related to comorbid conditions should be provided, including dietitians, psychologists, and bariatric surgeons.

### 6.3 Scope of long‑term follow‑up

During follow‑up, the following parameters should be regularly assessed:

- Treatment outcomes related to weight reduction (percentage of excess weight loss [%EWL], percent‑ age of BMI loss [%EBMIL], %TWL);
- Nutritional status and potential dietary deficiencies;
- Eating habits, along with qualitative and quantitative assessment of the diet;
- Resolution of obesity‑related diseases and adjustments to their treatment—diabetes remission criteria should follow international diabetic society guidelines (complete remission, partial remission, long‑term remission);
- Monitoring for new issues, distant complications, or other diseases;
- Laboratory tests, including:
- complete blood count;
- fasting glucose (HbA1c for diabetic patients);
- lipid profile;
- iron metabolism (ferritin, transferrin, iron);
- liver enzymes;
- renal function (urea, creatinine);
- calcium, magnesium, parathyroid hormone levels, bone alkaline phosphatase fraction;
- protein, albumin, and prealbumin levels;
- vitamin B12 and D3 levels;
- Assessment of issues specific to the surgical method;
- An endoscopic examination of the upper gastro‑ intestinal tract should be performed after bariat‑ ric surgery for all patients with GERD symptoms. Patients after SG or OAGB should undergo this ex‑ amination 1 year after surgery, and every 3–5 years thereafter, to exclude Barrett esophagus.^103^^,^^104^^,^^105^^,^^106^

### 6.4 Long‑term follow‑up schedule

Multidisciplinary follow‑up after MBS is essential for achieving optimal, long‑term treatment outcomes and ensuring patient safety. Each bariatric center should develop its own long‑term follow‑up system based on experience and avail‑ able resources. The frequency of follow‑up vis‑ its depends on the type of surgery, the rate of weight loss, and potential health problems after the procedure.

From the perspective of long‑term treatment outomes, the most important follow‑up visits are those scheduled during the first year postsurgery. Between 4 and 6 visits are recommended during this period, including medical consultations, as well as dietary and psychological counseling.

We recommend the following follow‑up schedule:

- 1, 3, 6, and 12 months after surgery;
- Thereafter once a year.

Follow‑up at the bariatric center can be carried out by an internist. Two years after surgery, ongoing care should be provided by primary health care services. If complications requiring surgical treatment are suspected, a consultation with a bariatric surgeon is recommended.

To increase attendance at follow‑up vis‑ its, the center should implement a reminder system to notify patients about upcoming appointments.^23^^,^^54^^,^^107^^,^^108^^,^^109^^,^^110^

### 6.5 Support groups

Numerous studies have shown a significant and positive impact of participation in support groups and associations for patients undergoing MBS, both pre‑ and postoperatively. Participation in these groups leads to increased knowledge and awareness about treatment, which can determine proper preparation for surgery and contribute to better postoperative outcomes, including weight loss, remission of obesity‑related diseases, quality of life improvements, fewer complications, and quicker recovery.

Many patients are aware of the existence and activities of support groups and associations, and participate actively in them even before deciding to undergo surgery. However, there is also a significant number of patients who are not familiar with these resources. Given the positive impact of patient involvement in support groups on treatment outcomes, it is recommended that all bar‑ iatric surgery patients be encouraged to maintain regular contact with such institutions, which of‑ ten operate in conjunction with surgical centers and are widely active on social media.

In Poland, there is a dynamic development of bariatric patient support groups on social media. The advantage of this phenomenon is the easy access to information and a sense of community, which may motivate patients to adhere to recommendations and maintain a healthy lifestyle. How‑ ever, there is a potential risk of unverified information or unprofessional advice, which could negatively affect treatment outcomes in extreme cases. One of the tasks for multidisciplinary teams is to verify the information patients obtain from social media.^111^^,^^112^^,^^113^^,^^114^

## 7. REVISIONAL SURGERIES

Revisional surgery is a procedure that corrects the failure or complication of previous surgical treatment. A patient qualifies for revisional surgery only when other treatment methods have failed. Indications for revision surgery in the treatment of obesity include:

- Bariatric failure;
- Metabolic failure;
- Long‑term complications that impair the patient’s daily functioning.

A new terminology has been proposed, recommending the use of the terms recurrence of excess body weight and lack of surgical response to describe failure of MBS so as to avoid attributing the failure solely to the patient. It is important to distinguish between these 2 conditions as separate entities.

The most common indications for revisional surgery are weight regain and recurrence of comorbidities, which comprise two‑thirds of all cases.

In patients with recurrence of excess weight, failure is caused by behavioral, metabolic, and / or genetic factors that influence long‑term treatment results. Late surgical complications that affect the efficacy of the primary procedure can also contribute to this outcome. For patients who did not respond to the surgical treatment, the cause may be

a biological or genetic predisposition that leads to early resistance to treatment.

For these reasons, revision procedures can be divided into 3 types:

- Conversion—changing the primary bariatric procedure due to its nonfunctionality;
- Corrective—modifying an unwanted effect of the primary bariatric procedure;
- Reversal—restoring the original anatomy of the gastrointestinal tract after the primary procedure due to poor tolerance or an unresolved complication.

Corrective procedures are usually planned to optimize the effectiveness of previous surgeries, while conversion procedures often introduce additional therapeutic mechanisms.

When qualifying a patient for revisional surgery, consideration should be given to the possibility of performing minimally invasive procedures, including endoscopic approaches.

When considering surgical conversion as a revisional procedure, alongside the direct reasons for qualification, clinical indications for the primary metabolic procedure and the surgeon’s skillset should also be taken into account.

It is unclear which revisional procedure is optimal. In all cases, qualification for surgical treatment shouldbe based on an individual case analysis and involve a multidisciplinary team (bariatric team). 

The most frequently performed revision procedures, depending on the primary procedure, are:

- AGB—dysfunction of the band or distant com‑ plications related to its presence (erosions, migrations) require removal of the device and conversion, usually to SG or RYGB.
- VBG—recurrence of obesity or dysfunction of the band usually requires conversion to RYGB or SG.
- SG—in the case of GERD or functional problems related to the sleeve (ie, stenosis, vomiting), con‑ version to RYGB is preferred. If the primary indication is weight recurrence or a lack of response without functional issues, options include RYGB, OAGB, BPD‑DS, or SADI‑S.
- RYGB—the presence of a gastro‑gastrostomy fistula, pouch dilation (>6 cm in length or >5 cm in width), or excessively wide gastrojejunostomy (>2 cm) as a cause of weight recurrence should be corrected by reconstructing the pouch (also endoscopically) or re‑establishing the anastomosis. Another option for correction is distalization of the intestinal anastomosis to reduce the overall length of the common limb.
- OAGB—in the case of GERD, conversion to RYGB is preferred. If the primary indication is weight recurrence or a lack of response with‑ out functional problems, options include RYGB, SADI‑S, or SG.

Literature data indicate that revisional surgeries, as compared with primary surgeries, show lower effectiveness in both weight reduction and the resolution of comorbidities. They are also associated with a significantly higher perioperative risk.

Qualification for revision surgeries should be based on a multidisciplinary team assessment, and the surgical procedures should be performed in highly specialized centers with appropriate infrastructure and experience.^71^^,^^115^^,^^116^,^117^,^118^,^119^,^120^

## 8. THE ROLE OF PHARMACOTHERAPY IN METABOLIC AND BARIATRIC SURGERY

The following medications are registered in Poland for the treatment of obesity:

- Phentermine + topiramate in an extended‑re‑ lease capsule (7.5 mg + 46 mg; 11.25 mg + 69 mg; 15 mg + 92 mg)—centrally‑acting appetite suppressant + antiepileptic agent;
- Orlistat (120 mg)—a specific and long‑acting lipase inhibitor produced in the gastrointestinal tract;
- Bupropion + naltrexone (90 mg + 8 mg)—a dopamine and norepinephrine reuptake inhibitor + µ‑opioid receptor antagonist;
- Liraglutide (3 mg)—a human GLP‑1 analog;
- Semaglutide (2.4 mg)—a long‑acting human GLP‑1 analog;
- Tirzepatide (5 mg, 10 mg, 15 mg)—a long‑acting agonist of gastric inhibitory polypeptide (GIP) and GLP‑1 receptors.

Research results have shown that their use leads to weight loss averaging from 6% to 23% of total body weight, depending on the medication used. Long‑term observations (as of now for one of the medications) have also shown a beneficial effect of pharmacological treatment of obesity on reducing the frequency of major cardio‑ vascular events.

Given the above and the fact that pharmaco‑ logical treatment of obesity carries a lower risk than surgical treatment, it is recommended that patients be informed about the possibility of using pharmacological treatment as the first line of therapy before being qualified for MBS. Pharmacological treatment, similarly to surgical treatment, should include nutritional education, psychological support, and physical rehabilitation. Patients should also be informed that pharmaco‑logical treatment is chronic (permanent), as otherwise weight regain will occur.

In the cases where pharmacological treatment is ineffective (weight loss below 5% after 3 months of medication use), switching the medication is recommended. If further ineffectiveness occurs, surgical treatment should be considered. Given that preoperative weight loss is one of the key elements for perioperative safety, as it reduces the incidence of early postoperative complications, pharmaco‑ logical treatment of obesity should be considered for patients who do not achieve the desired weight loss through nonpharmacological methods (neo‑ adjuvant treatment).

In the case of obesity recurrence or insufficient weight loss after MBS, and excluding surgical causes of the situation, pharmacological treatment should be recommended before considering revision surgery.

If general anesthesia is required, it is recommended to discontinue medications that are GLP‑1 and GIP receptor agonists according to their ad‑ ministration schedule. The last dose of short‑acting medications should be taken no later than on the day before the surgery, and long‑acting medications, no later than 1 week before the surgery. If adverse effects, such as nausea, vomiting, bloating, or abdominal pain, persist despite discontinuation of these medications, surgery should be postponed until these symptoms resolve. If surgery is urgent or expedited, the protocol typical of a “full stomach” should be followed. With respect to medications containing naltrexone, an opioid recep‑ tor antagonist, it is recommended to stop taking them at least 48 hours before the planned surgery. Therapeutic actions should be reflected in the patient’s individual medical documentation. Patients should also be informed that, at present, pharmacological treatment of obesity with these medications is not reimbursed by public funds at any stage of the therapy.^121^^,^^122^

## 9. EXCEPTIONAL CIRCUMSTANCES

In certain circumstances, any diagnostic and therapeutic decisions regarding obesity treatment should be made individually, based on the conciliar decisions of an appropriately expanded therapeutic team and the patient’s specific needs.

### 9.1. Gynecological and obstetric care

In women of reproductive age (up to 50 years) with an intact uterus, who undergo bariatric treatment, contraception should be started preoperatively and continued for 12–18 months after surgery. A gynecological consultation before surgery regarding the choice of the contraception method is advised, as preoperative counseling has been proven to increase the likelihood of proper use of contraception postoperatively. Due to possible absorption disturbances of both estrogenic and progestogenic components in oral contraceptives, especially after RYGB, intrauterine devices and subcutaneous implants releasing progestogens, or possibly barrier methods, are recommended.

Due to a higher frequency of menstrual disturbances in women with obesity and the inability to unequivocally assess the phase of the cycle, it is recommended to measure β‑human chorionic gonadotropin levels before surgery in all women of reproductive age with an intact uterus.

Pregnancy is contraindicated during the rapid weight loss phase after surgery due to an increased risk of miscarriage, hypoglycemia, nutrient deficiencies, malnutrition of the fetus, and consequently intrauterine growth restriction. The minimum period between surgery and attempts to conceive is considered 12 months, until weight stabilization, ideally 12–18 months, due to possible vitamin and micronutrient deficiencies. Earlier conception can be individually considered in the cases of advanced maternal age or reduced ovarian reserve. Due to a higher risk of nutritional deficiencies after RYGB than after SG, and consequently a greater risk of intrauterine growth restriction of the fetus, SG appears to be a safer treatment option for women of reproductive age, but literature data on this topic are limited.

MBS leads to a significant reduction in the risk of gestational diabetes, pregnancy‑induced hypertension, preterm birth, and the birth of a new‑ born with high birth weight. At the same time, it increases the risk of intrauterine growth restriction, low birth weight, nutritional deficiencies, and deficiency anemia in the pregnant woman. Every pregnancy in a patient who has undergone MBS should be treated as high‑risk and managed by an obstetrician‑gynecologist experienced in the treatment of patients after MBS. The delivery should ideally take place in a center with a referral level greater than level I. Continuous vita‑ min and micronutrient supplementation is necessary, and regular monitoring of at least peripheral blood count, ferritin concentration, and vita‑ min B12 levels is required.

The standard gestational diabetes diagnostic test, the 75‑g oral glucose tolerance test, should not be used in patients after bariatric surgery due to a risk of late postprandial syndrome and hypoglycemia, which may cause fetal distress. The recommended alternative diagnostic method for gestational diabetes is a weekly evaluation of fasting blood glucose levels and levels after main meals, or a continuous 7‑day transdermal glucose monitoring. This should be conducted twice, around the eighth–tenth week of pregnancy, and again between weeks 24 and 28. The evaluation may be complemented by the measurement of HbA_1c_ levels.

A history of MBS is not an indication for cesarean delivery. Breastfeeding is recommended, and no nutritional deficiencies of breast milk are observed in patients after bariatric surgeries, pro‑ vided that potential nutritional deficiencies are addressed.^55^^,^^123^^,^^124^^,^^125^^,^^126^

### 9.2. Inflammatory bowel disease

There are no absolute contraindications for qualifying patients diagnosed with inflammatory bowel disease (IBD) for bariatric treatment, and the results in this group of patients are comparable with those observed in individuals with‑ out IBD. A close collaboration between the bariatric team and a gastroenterologist is crucial when qualifying IBD patients for MBS.

Factors that need to be considered during qualification include disease activity, medication use, nutritional status, and the potential impact of obesity treatment on IBD symptoms, as some studies suggested a correlation between MBS and the development IBD. Specific dietary needs and specialized gastroenterological care for such patients should also be considered.

The obesity treatment scheme for patients with IBD should be similar to that followed in the case of patients without IBD, starting with behavioral interventions, through the introduction of antiobesity medications, and ending with MBS.

The choice of a surgical treatment method for patients with IBD is not straightforward, although there are reports suggesting a higher incidence of complications after RYGB in patients diagnosed with IBD.

During the perioperative period, strict monitoring of IBD activity is necessary, and postoperative care should be tailored individually to each patient. Regular bariatric and gastroenterological follow‑up is crucial in managing additional problems arising from IBD, and should be included in the postoperative care plan.^127^^,^^128^^,^^129^^,^^130^^,^^131^

### 9.3. Celiac disease

Up to 10% of patients with celiac disease may present symptoms of pathological obesity. There are no absolute contraindications to qualifying patients with diagnosed celiac disease for bariatric treatment, although there is no clear scientific evidence regarding the impact of bariatric surgery on clinical treatment outcomes. Close collaboration between the bariatric team and a gastroenterologist is crucial.

Patients with celiac disease who are qualified for MBS should be evaluated for nutritional status (malnutrition, sarcopenia, micronutrient deficiencies, overweight, obesity) at diagnosis and then at regular intervals (at least once a year). Celiac patients are at a risk of additional nutrition‑ al deficiencies due to the metabolic effects of surgery. Regular bariatric and gastroenterological follow‑up is essential in managing additional issues arising from celiac disease, and should be included in the postoperative care plan.^132^^,^^133^

### 9.4. Oncological patients

Obesity is one of the major risk factors for the development of malignancy, including esophageal, breast, colorectal, endometrial, gastric, pancreatic, ovarian, and liver cancers. Numerous scientific studies indicate that MBS can contribute to a reduction in the risk of developing these cancers. Given the increasing number of cancer patients and the rising proportion of individuals eligible for bariatric treatment, clinical decisions regarding qualification for surgery in this group of patients are expected to be made more frequently. It has been previously assumed that a specific amount of time must pass between the completion of oncological treatment and MBS. However, it is now suggested that each patient previously treated for cancer be qualified individually, based on the decision of the clinical oncologist. There is no exact time frame specified, but the qualification should consider the type and stage of the previously treated malignancy, the patient’s overall condition, and gastrointestinal status (type of previous oncological surgeries). Various calculators assessing the risk of cancer recurrence can be used, and it is essential to take into account that bariatric surgery may hinder or even prevent the initiation of treatment in the case of recurrence. Indisputably, the presence of active cancer is an absolute contraindication to bariatric surgery, regardless of the type of malignancy.^16^^,^^20^^,^^134^^,^^135^^,^^136^^,^^137^.

## 10. PLASTIC SURGERY AFTER METABOLIC AND BARIATRIC SURGERY–INDUCED WEIGHT LOSS

Body contouring surgery should be considered in patients whose excessive weight loss has reached at least:

- 60% in the patients with no preoperative complications; or
- 40% in the patients with preoperative complications, particularly metabolic ones; or
- When excess skin causes skin conditions, functional disturbances, or significantly reduces the patient’s quality of life, regardless of the degree of postoperative weight loss.

Patients eligible for body contouring surgery should meet the following criteria:

- Stabilization of body weight within approximately 2% for 3 months prior to the surgery;
- No identified nutritional deficiencies.

Body contouring surgery is contraindicated in patients who:

- Smoke or are addicted to psychoactive substances;
- Have untreated mental health issues despite therapy and pharmacological treatment.

Qualification for plastic surgery should be made by a multidisciplinary team based on qualification criteria, an assessment of the patient’s general condition, and the absence of contraindications to the procedure.

## 11. GUIDELINES SERVING AS THE BASIS FOR THE ABOVEMENTIONED RECOMMENDATIONS

2005 – European Association for Endoscopic Surgery (EAES)

2005 – Recommendations regarding obesity surgery

2009 – Guidelines of the Chapter for Metabolic and Bariatric Surgery of the Association of Polish Surgeons

2010 – American Association of Clinical Endocrinologist (AACE), The Obesity Society (TOS), American Society for Metabolic and Bariatric Surgery (ASMBS)

2011 – International Diabetes Federation (IDF) 2011 – Association of Scientific Medical Societies in Germany (AWMF)

2012 – ASMBS Pediatric Committee Best Practice Guidelines

2013 – Interdisciplinary European Guidelines on Metabolic and Bariatric Surgery

2013 – European Association for the Study of Obesity (EASO), IFSO‑EC

2014 – National Institutes of Health (NIH) 2014 – American Diabetes Federation (ADA) 2014 – Diabetes Poland

2014 – Cochrane Collaboration

2015 – European Guidelines for Obesity Management in Adults

2016 – Treatment Algorithm for Type 2 Diabetes: A Joint Statement by International Diabetes Organizations

2018 – ASMBS Pediatric Metabolic And Bariatric Surgery Guidelines, 2018

2020 – Clinical practice guidelines of the European Association for Endoscopic Surgery (EAES) on Bariatric Surgery: update 2020 endorsed by IFSO‑EC, EASO, and ESPCOP

2020 – British Obesity and Metabolic Surgery Society Guidelines on Perioperative And Postoperative Biochemical Monitoring and Micronutrient Replacement for Patients Undergoing Bariatric Surgery

2021 – Guidelines for Perioperative Care in Bar‑ iatric Surgery: Enhanced Recovery After Surgery (ERAS) Society Recommendations: a 2021 update 2022 – 2022 American Society of Metabolic and Bariatric Surgery (ASMBS) and International Federation for the Surgery of Obesity and Metabolic Disorders (IFSO)

2024 – Current recommendations for procedure selection in class I and II obesity developed by an expert modified Delphi consensus
